# Effectiveness of Nurse-Led Stroke Rehabilitation on Awareness, Activities of Daily Living and Coping in Stroke Patients at a Tertiary Care Hospital in India

**DOI:** 10.7759/cureus.72843

**Published:** 2024-11-01

**Authors:** N Mangalabarathi, Bamini Devi, Kanniammal Chinnathambi, Nirmala C

**Affiliations:** 1 Nursing, SRM College of Nursing, SRM Institute of Science and Technology, Chennai, IND; 2 General Medicine, SRM Medical College Hospital and Research Centre, SRM Institute of Science and Technology, Chennai, IND

**Keywords:** activities of daily living (adl), coping skills, inpatient rehabilitation, mortality rate in icu, nurse led stroke rehabilitation, physical disability, post-stroke rehabilitation, stroke, stroke awareness, stroke complications

## Abstract

Introduction

Stroke is a major global cause of mortality and disability, with many survivors lacking awareness. Nurses play a pivotal role in the early identification and management of symptoms with regard to stroke. The present study aimed to assess the effectiveness of stroke rehabilitation on awareness, activity of daily living (ADL) and coping among stroke patients.

Methods

A quasi-experimental approach was used, with 60 stroke patients (30 patients in the study and 30 patients in the control group) selected using a non-probability purposive sampling technique. The pre-test on stroke awareness by the stroke knowledge assessment tool, ADL was assessed by the Barthel Index, and coping was assessed through the Brief-COPE (Coping Orientation to Problems Experienced) scale among stroke patients. The investigator implemented stroke rehabilitation for study group participants and routine was followed for control group patients. Nurse-led stroke rehabilitation consists of Day 1 lecture on the causes, medication adherence, post-stroke complications, and home care following a stroke; Day 2: Tailored to individual needs - demonstration of meeting ADL such as bathing, toileting, grooming, and feeding; Day 3: Demonstration of active and passive range of motion (ROM) exercises; Days 4-7: Supervised practice sessions on meeting daily activities as well as active and passive exercises. Daily practices last 40 minutes once a day was carried out. Post-test was conducted on the 30^th^ day at neurology OPD during a re-visit, on stroke awareness, ADL and coping. The data was organized and analyzed using descriptive and inferential statistics.

Results

The control group had a mean awareness of causes, symptoms, management, and prevention of complications of stroke with a mean score of 11.7 while the study group had 13.6, which was statistically significant at p < 0.05. ADL comparison between control and study groups revealed that the ADL mean score was 12.07 in the control group and 13.23 in the study group significant at p < 0.05. A significant disparity in coping was found between the control group (30.3) and the study group (35.03), which was statistically significant at p < 0.01.

Conclusion

The study reveals that stroke rehabilitation led by nurses is more effective for stroke patients in improving their coping abilities, ADLs, and awareness.

## Introduction

Stroke is the second leading cause of death globally and a major contributor to disability [1]. Over the last 17 years, the lifetime risk of experiencing a stroke has increased by 50%, with approximately one in four people likely to face one. Ischemic stroke is the predominant form, aiming to heal neurological injuries and improve blood circulation to the brain. The aftermath of a stroke impacts not only the individual but also their families, healthcare infrastructure, and the broader economic landscape [2]. The challenges faced in recovery and rehabilitation can ripple through various aspects of life, creating a complex web of consequences that extend beyond the patient. Understanding these implications is crucial for developing effective support systems and policies that address the needs of all stakeholders involved in post-stroke care. Effective stroke prevention, recovery, and treatment hinge on the enhancement of both preclinical and clinical care [3]. The American Heart Association reports that there are 9.4 million stroke cases globally, accounting for 3.3% of the population. The worldwide occurrence of ischemic stroke is projected to increase to 89.32 per 100,000 by 2030, with an anticipated annual percentage change of 0.89. For every 100,000 individuals, the mortality rate and disability-adjusted life years should decrease to 18.28 and 500.37, respectively [4].

A review of 16 studies on 2072 stroke patients indicated that nurse-led programs decreased motor impairment better than traditional therapy. Nurse-led interventions raised Barthel index (BI) scores and lowered modified Rankin scale (mRS) scores above 2. Nurse-led programs manage and monitor stroke patients better, which may improve motor disability [5]. Acute ischemic stroke patients improved significantly following seven days of rehabilitation nursing and therapist-led treatment. Although the nursing program matched the effectiveness of therapist-led treatment, the lack of conclusive findings highlights the need for further investigation [6]. Nurses provide aftercare for stroke patients, leading to long-term psychological outcomes. Compared to normal treatment, quality-adjusted life years (QALYs) and social participation improved significantly. The average cost of stroke aftercare was €91 per person, €1208 more than typical treatment. Initial cost-effectiveness assessments revealed an incremental cost-effectiveness ratio of €24,679 per QALY gained, with a 64% likelihood at €50,000 willingness to pay [7].

Stroke-related awareness and preventive behaviors among stroke patients in Taizhou, China

A total of 156 patients completed questionnaires, and a generalized linear model was used to investigate factors influencing preventative behaviors. Results showed that 36.5% had a strong awareness of stroke warning signals, 40.4% had a good understanding of risk factors, and 57.7% demonstrated effective stroke preventive practices. A positive correlation was found between higher stroke-related information scores and better preventative actions. The study also found that individuals aged 60 or older, females, those engaging in physical activities, or those without underlying disorders were associated with better preventive measures. Overall, participants had insufficient stroke-related information and inadequate practice [8]. An integrative review of 169 papers on stroke awareness found significant variability in identifying risk factors for stroke, with percentages ranging from 18% to 94% for open-ended questions and 42% to 97% for closed questions. Participants' ability to identify a single symptom varied from 25% to 72% for open-ended questions and 95% to 100% for closed questions. In the event of a suspected stroke, 53% to 98% of individuals indicated prompt contact with emergency medical services [9].

Effectiveness of the stroke empowerment program in improving stroke awareness among caregivers of stroke survivors

The result showed that the mean pre-test awareness score for the study group was 6.35, while the mean score for the control group was 6.89. The average score for post-test awareness was 11.46, 11.68, and 11.52 in the study group, and 7.42, 7.58, and 7.48 in the control group for post-tests I, II, and III, respectively. The average score in the study group caregivers was higher than that of the control group caregivers during post-tests, and this difference was statistically significant at a significance level of p < 0.01 [10].

A cross-sectional study to evaluate the dependency in activities of daily living (ADL) and identify its predictors

The objective of this study was to determine the prevalence of reliance on ADL and identify the factors that predict it among the elderly population living in rural areas. The findings revealed that the average age of the individuals involved in the study was 68.31 ± 7.9 years. ADL dependency was present in 46.3% of the individuals, with most of them showing a low to moderate level of dependence. Merely 2.5% of the participants indicated a significant level of dependence on ADL. The average ADL score was 94.47 ± 8.98. Age, educational level, family stress, personal history, and co-morbidities were identified as independent predictors of ADL dependency by multivariate logistic regression analysis [11].

A quantitative study was conducted to assess the stress and coping among family caregivers among 60 stroke victims, which was recruited by purposive sampling technique. Stress was assessed through a modified version of the perceived stress scale and a questionnaire on coping strategies. The results indicated that 98% of families with stroke patients experienced a moderate degree of stress, while 2% of families experienced a low level of stress. Additionally, it was discovered that 96% of them exhibited a moderate level of coping, whereas 4% showed a high level of coping [12]. Stroke nurses are crucial in the care of stroke patients, providing specific monitoring and attention during the acute phase of the disease to avoid complications like cerebral edema or rebleeding. They receive training in the early detection of stroke and neurological changes, which facilitates the implementation of specific therapies [13].

The objective of the present study was to assess the level of stroke awareness, ADL, and coping among patients with stroke in the control and study group and to evaluate the effectiveness of nurse-led stroke rehabilitation of stroke awareness, ADL, and coping among patients with stroke in the control and study group. Nurse-led stroke rehabilitation is a patient-centered process in which a stroke nurse focuses on the patient’s functional ability and quality of life through physical therapy, counseling, and education. As part of this intervention, stroke prevention awareness education is also included. The hypothesis RH-1 posits that patients engaged in nurse-led stroke rehabilitation would exhibit substantial enhancements in stroke awareness, ADL, and coping mechanisms compared to those who do not participate.

Stroke nurses also support the early phase of rehabilitation by helping patients regain important abilities and preventing further complications. They educate patients and their families on stroke treatment and offer advice on how to reduce stroke recurrence by changing their lifestyle. By coordinating care with a multidisciplinary team, stroke rehabilitation nurses ensure a holistic approach to therapy and emotionally support patients and their families during the healing process. They develop individualized care plans targeting mobility, speech, and daily living tasks, aiding patients in their everyday activities, providing education on stroke management, and facilitating care coordination between healthcare professionals. Their primary focus is on preventing secondary problems and recurrent strokes by providing information and implementing lifestyle changes, ultimately improving patients' quality of life and independence [14].

Taking into consideration the information presented above, the investigator was interested in finding the effectiveness of nurse-led stroke rehabilitation on stroke awareness, ADL, and coping among patients with stroke.

## Materials and methods

This study was an interventional study that was conducted at SRM Medical College Hospital and Research Centre, Kattankulathur, Chennai, India. Using a non-probability purposive sampling approach, 60 stroke patients (30 in the study group and 30 in the control group) were selected as per the inclusion criteria such as patients who are conscious and oriented to place, time, and person; patients who are diagnosed as having a stroke with an mRS score of less than 3, which indicates moderate disability; age between 35 and 60 years (in India, a significant portion of stroke occurs among individuals under 40 years of age); patients who were diagnosed as hemorrhagic or ischemic stroke; patients who are hemodynamically stable; and patients who are able to understand Tamil or English. The study participant in the control group received routine care rendered by the hospital which consisted of usual nursing care by stroke nurses, physiotherapy, and occupational therapy whereas the study group participants received nurse-led stroke rehabilitation interventions. At the end of the study, control group patients were given post-stroke rehabilitation education. The purpose of this is to assess the level of awareness on causes, symptoms, management, and prevention of complications of stroke, ADL, and coping with post-stroke disability among stroke patients, and to evaluate the effectiveness of nurse-led stroke rehabilitation on stroke awareness, ADL and coping in the control and study group.

**Figure 1:**
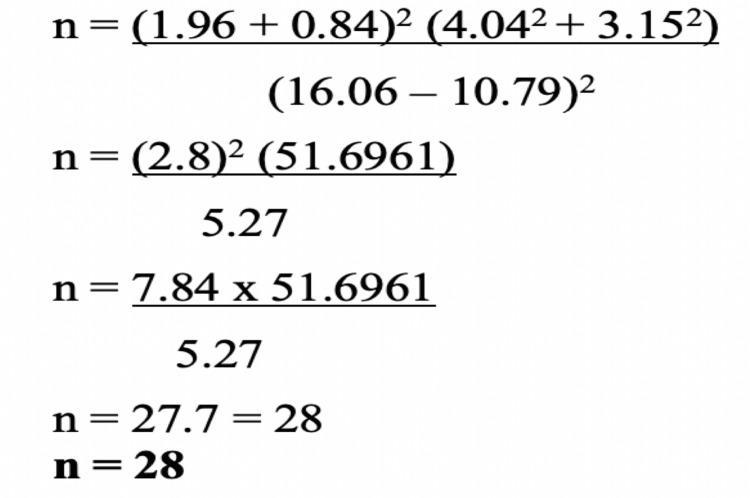
Sample Size Calculation The calculated sample size is 28. Therefore, we considered 28 samples from each study and control group. In order to account for attrition, the sample size was rounded up to 30 in each study and control group.

Considering the prevalence of stroke from the study by Kamalakannan et al. [15,16] the sample size was calculated and mentioned in Figure 1.

Written consent was obtained from the patients and then allocated to the study and control group by purposive sampling technique. Demographic and clinical variables were collected with self-structured questions. The pre-test on stroke awareness by the stroke knowledge assessment tool [16], ADL was assessed by the BI [17], and coping was assessed through the Brief-COPE scale [18] among stroke patients. Nurse-led stroke rehabilitation is implemented through one-to-one laptop-assisted teaching for patients following stroke through lecture cum demonstration method, which consists of 40 minutes of inpatient teaching sessions for three days, which include Day 1: lecture on causes, medication adherence, management and prevention of post-stroke complication and home care management of stroke; Day 2: educating on meeting day-to-day activities such as bathing, toilet, grooming, feeding; Day 3: demonstration of active and passive exercise; Days 4 to 7: supervised practice on meeting day-to-day activities, active and passive exercise.

Following the completion of the intervention, a post-test was conducted on the 30th day using the same tool. Four patients from the control group and three patients from the study group did not attend the follow-up visit during the post-test because of their obligations. As a result, they were excluded from the study.

The data collected from the 60 stroke patients on stroke awareness, ADL, and coping was tabulated and analyzed by using IBM SPSS Statistics for Windows, Version 23 (Released 2017; IBM Corp., Armonk, New York, United States).

## Results

Demographic variables of stroke patients

This study concluded with a total of 60 patients with stroke admitted to the neurology ward and neurology outpatient department (OPD). Table 1 reveals the demographic variables of stroke patients. With regard to the age in the control group, 12 patients (40%) were between the age of 30-40 years and above 50 years respectively, whereas in the study group 21 patients (70%), were above the age of 50 years. In relation to gender, 24 (80%) of the patients in the control group and 16 patients (53%) in the study group were male. When focusing on educational status, in the control group, 20 patients (67%) were illiterate and in the study group, 25 patients (83%) were illiterate. When assessing the marital status, in the control group, 26 patients (87%), and in the study group, 21 patients (70%) were married. During the evaluation of employment status, in the control group, the majority of the patients, 18 (60%), and 23 (77%) in the study group were employed. Regarding the monthly income, in the control group, the majority of the patients, 13 (43%) and 11 (37%) in the study group earned between 10,241 and 20,481 Rupees. In terms of the family type within the control group, 29 patients (97%) and 18 patients (60%) in the study group belong to a nuclear family.

**Table 1:** Frequency and Percentage Distribution of Demographic Variables Among Patients With Stroke in Control and Study Groups

S. No	Demographic Variables	Control Group		Study Group	
		No.	Percentage	No.	Percentage
1	Age				
	30-40 years	12	40	0	0
	41-50 years	6	20	9	30
	Above 50 years	12	40	21	70
2	Gender				
	Male	24	80	14	47
	Female	6	20	16	53
3	Education				
	Professional	1	3	1	3
	Diploma	1	3	0	0
	Higher secondary school	7	23	2	7
	High school	1	3	2	7
	Illiterate	20	67	25	83
4	Marital Status				
	Married	26	87	21	70
	Unmarried	3	10	0	0
	Widower	1	3	9	30
5	Occupation				
	Employed	18	60	23	77
	Unemployed	12	40	7	23
6	Monthly Income				
	5120-7680	1	3	1	3
	7681-10,240	5	17	8	27
	10,241-20,481	13	43	11	37
	>20,482	0	0	8	27
	None	11	37	2	7
7	Type of Family				
	Joint family	0	0	10	33
	Nuclear family	29	97	18	60
	Extended family	1	3	2	7

Clinical variables of stroke patients

**Figure 2:**
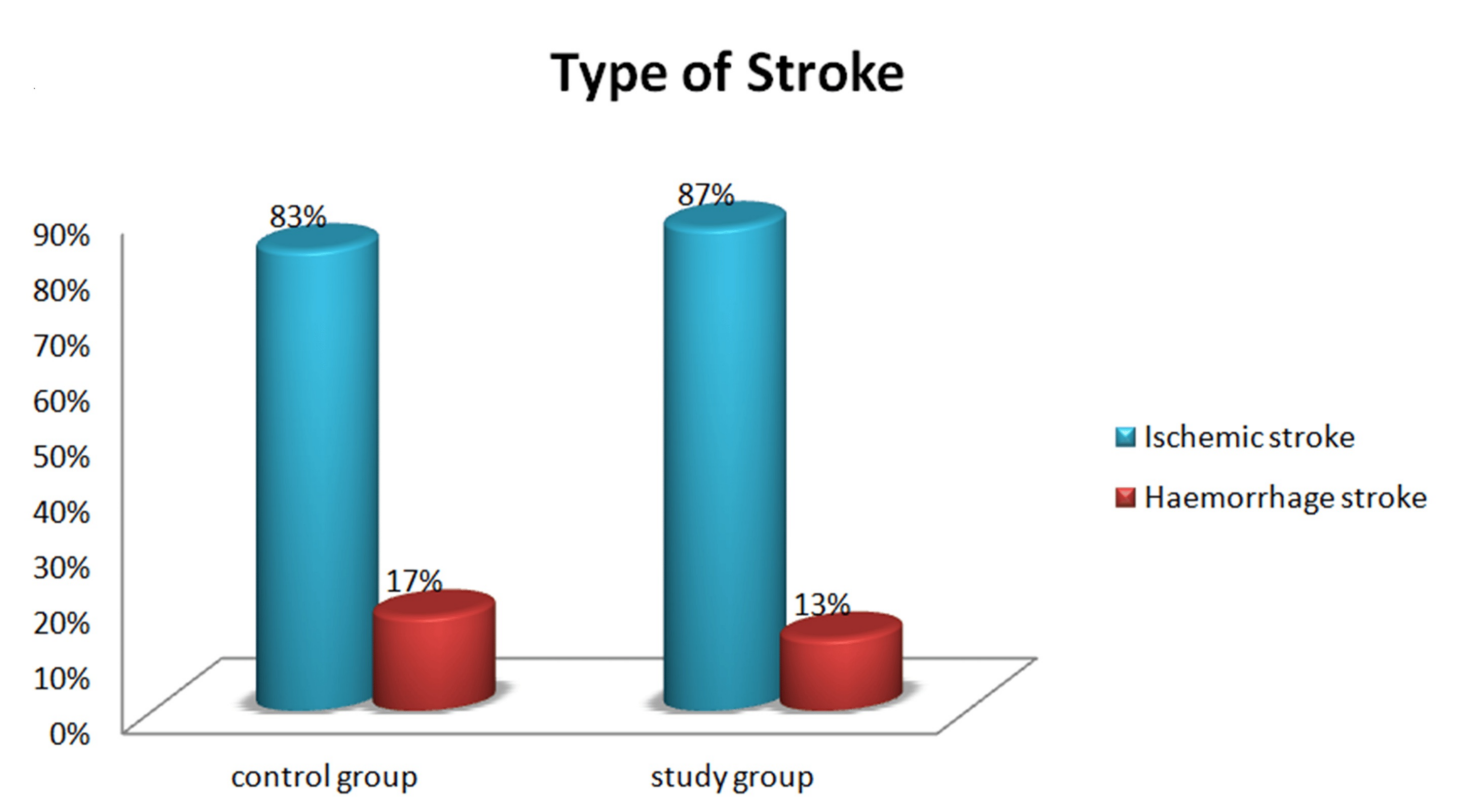
Percentage Distribution of Type of Stroke Among Patients With Stroke in Control and Study Groups

**Figure 3:**
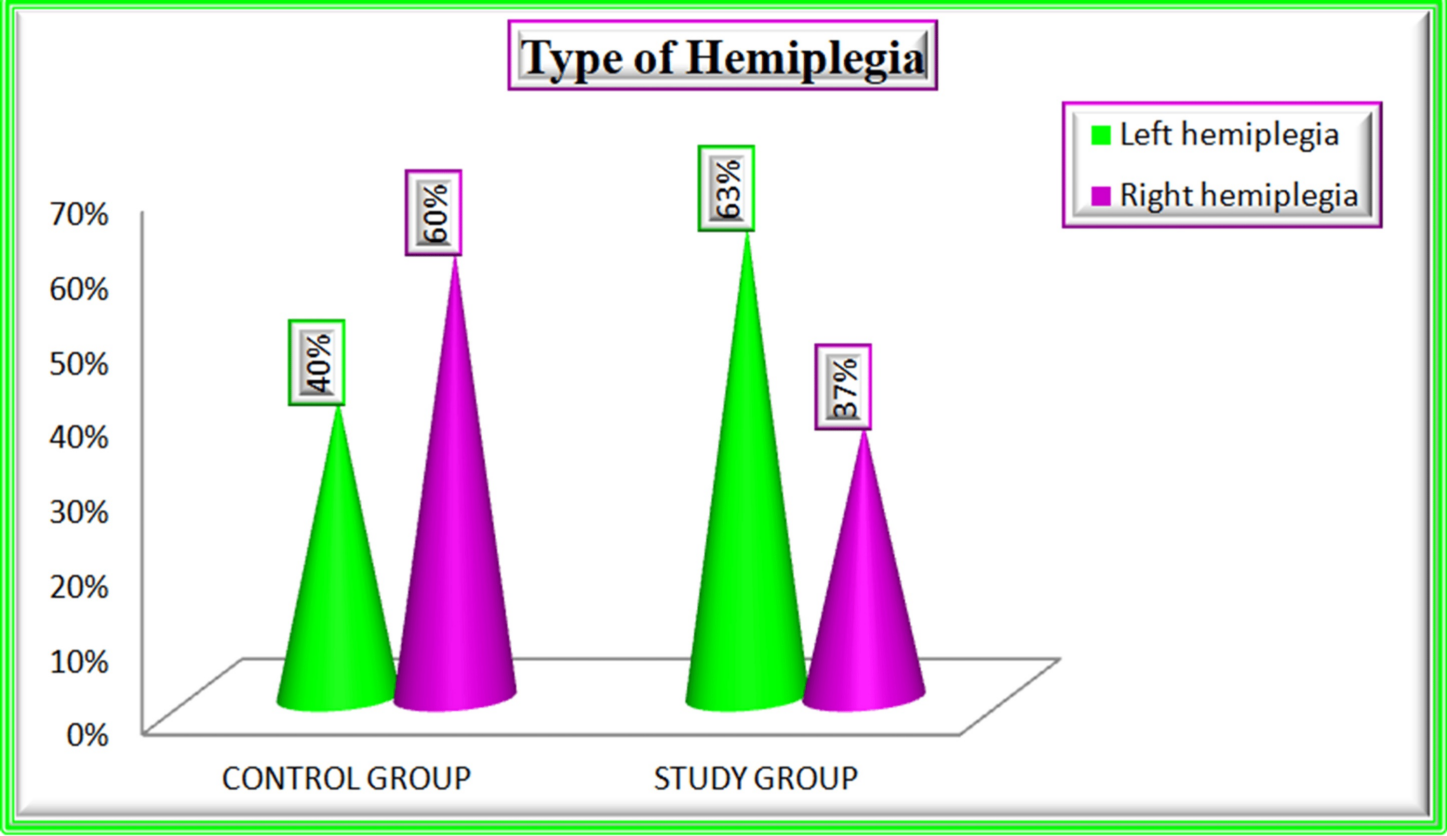
Percentage Distribution of Type of Hemiplegia Among Patients With Stroke in Control and Study Groups

**Figure 4:**
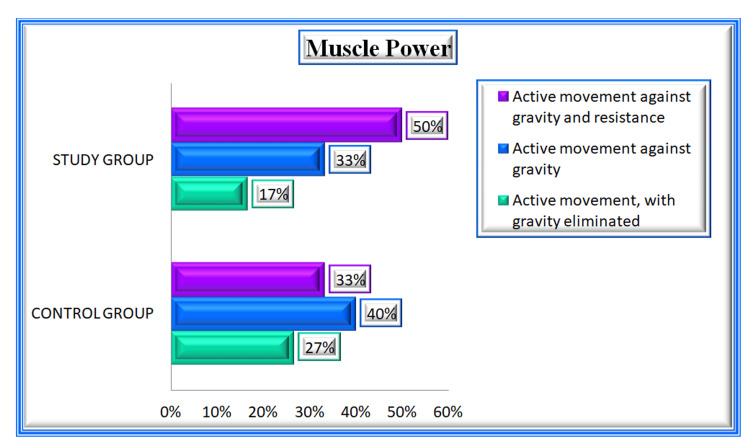
Percentage Distribution of Muscle Power Among Patients With Stroke in Control and Study Groups

The clinical variables of the study participants are tabulated in Table 2. Regarding the history of diabetes mellitus 21 patients (70%), in the control group, and 23 patients (77%) in the study group were diagnosed with diabetes. Twenty (67%) patients in the control group and 11 patients (37%) in the study group had a history of hypertension. During the assessment of stroke symptom onset, the control group 11 (37%) arrived at the ER within six hours, and the study group 14 (47%) arrived at the ER within six hours. Regarding the type of stroke in the control group 25 (83%) and in the study group 26 (87%) were affected by ischemic stroke and five (17%) in the control group, four (13%) in the study group were affected by hemorrhagic stroke (Figure 2). During an examination of hemiplegia types in the control group, 18 (60%) and in the study group, 19 (63%) patients were affected by left hemiplegia (Figure 3). Glasgow Coma Scale (GCS) score for the control group 19 (63%) patients scored 14 out of 15 and in the study group, 23 (77%) patients scored 13 out of 15. While checking the muscle power in the control group 12 (40%) patients achieved active movement against gravity and in the study group, 15 (50%) patients achieved active movement against gravity and resistance (muscle power - 3) (Figure 4). While checking the temperature in the control group 12 (40%) and 14 (47%) in the study group patients had high temperatures (38.0-38.5 degree Celsius). During the blood sugar monitoring in the control group, 21 patients (70%) and 20 patients (67%) had high blood sugar (161-200). On the assessment of swallowing difficulties in the control group, 16 (53%) patients had mild trouble swallowing solids whereas in the study group, 20 (67%) patients had significant difficulty swallowing solids, often requiring a soft diet.

**Table 2:** Frequency and Percentage Distribution of Clinical Variables Among Patients With Stroke in Control and Study Groups GCS: Glasgow Coma Scale

S. No	Clinical Variables	Control Group		Study Group	
		No.	Percentage	No.	Percentage
1	History of diabetes mellitus				
	Yes	21	70	23	77
	No	9	30	7	23
2	History of hypertension				
	Yes	20	67	11	37
	No	10	33	19	63
3	Onset of stroke symptoms admitted to stroke unit				
	Within 4 hrs	10	33	4	13
	Within 6 hrs	11	37	14	47
	Within 12 hrs	9	30	12	40
4	Types of stroke				
	Ischemic stroke	25	83	26	87
	Hemorrhage stroke	5	17	4	13
5	Type of hemiplegia				
	Left hemiplegia	12	40	19	63
	Right hemiplegia	18	60	11	37
6	GCS Score				
	13	11	37	23	77
	14	19	63	7	23
7	Muscle power				
	Active movement, with gravity eliminated	8	27	5	17
	Active movement against gravity	12	40	10	37
	Active movement against gravity and resistance	10	33	15	50
8	Temperature (in Celcius)				
	37.0-37.5	8	27	5	17
	37.5-38.0	10	33	11	37
	38.0-38.5	12	40	14	47
9	Blood sugar (in mg/dL)				
	<=120	4	13	3	10
	121-160	5	17	7	23
	161-200	21	70	20	67
10	Swallowing difficulty				
	Mild difficulty swallowing solids	16	53	10	33
	Significantly difficulty swallowing solids, often requires soft diet	14	47	20	67
11	Known history of cardiovascular disorders				
	Yes	10	33	13	43
	No	20	67	17	57
12	Family history of stroke				
	Yes	7	23	12	40
	No	23	77	18	60

Stroke awareness

**Figure 5:**
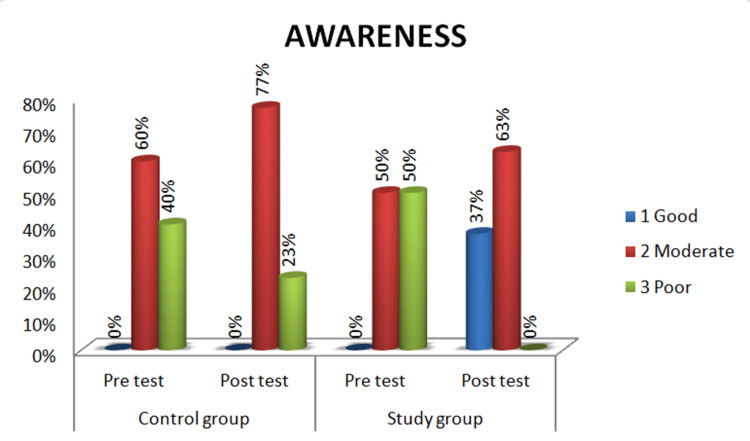
Percentage Distribution of Awareness Among Patients With Stroke in Control and Study Groups

In the control group, during the pre-test, the majority of patients, 18 (60.0%), had a moderate level of awareness about stroke, while 12 patients (40.0%) had a poor level of awareness. None of the patients had a good level of awareness about stroke. In the post-test, the majority of patients 21 (70.0%) had mild to moderate awareness about stroke, while nine (30.0%) had poor awareness. None of the patients showed good awareness of stroke. In the study group, the pre-test results showed that all 30 patients (100.0%) had poor awareness of stroke, whereas none of the patients had moderate or good awareness of stroke. In the post-test, the majority of patients, 20 (66.7%), showed moderate awareness of stroke. Additionally, 10 patients (33.3%) showed good awareness while none of them had poor awareness of stroke after implementing nurse-led stroke rehabilitation (Figure 5). The mean stroke awareness score during the post-test was 11.7 in the control group and 13.6 in the study group and the calculated p-value of 0.125 which was not statistically significant. However, the post-test mean score in the study group was higher when compared to the control group (Table 3).

**Table 3:** Comparison of Mean Stroke Awareness, ADL and Coping Between the Control and Study Groups Among Patients With Stroke ** indicates significant at 1% level; * indicates significant at 5% level; ADL: activity of daily living

Variables	Group	Control Group		Study Group		Mean Df	T-value	P-value
		Mean	SD	Mean	SD			
Awareness	Pre-test	10.4	6.97	10.7	5.16	0.3	0.189	0.850
	Post-test	11.7	3.41	13.6	5.77	1.9	1.55	0.125
ADL	Pre-test	10.1	6.8	10.2	5.1	0.100	0.064	0.9488
	Post-test	12.07	2.490	13.23	1.073	1.16	2.216	0.031*
Coping	Pre-test	20.54	3.42	21.8	3.7	1.260	1.370	0.1761
	Post-test	30.3	3.098	35.03	1.712	4.73	6.914	0.000**

ADL

**Figure 6:**
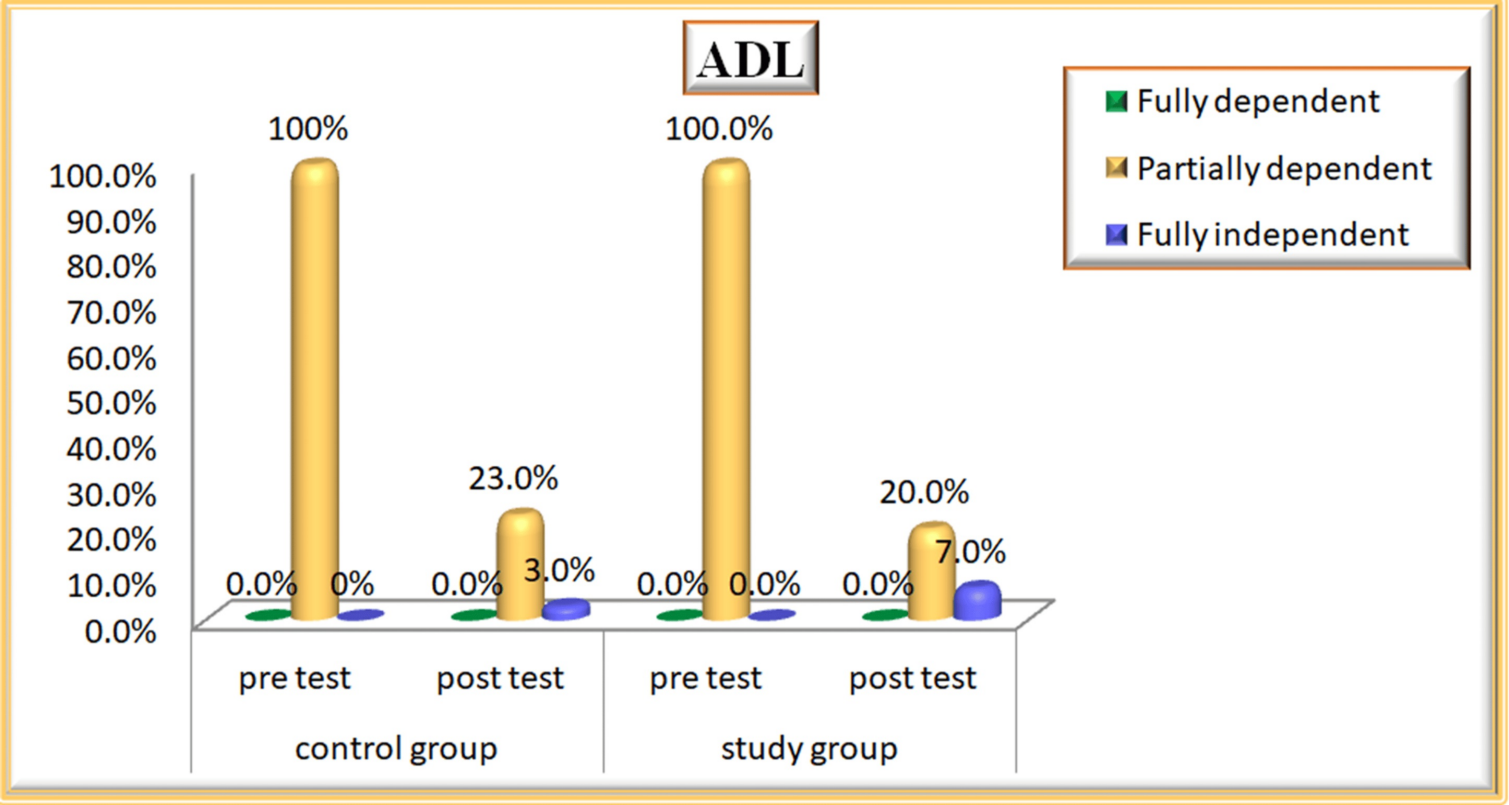
Percentage Distribution of ADL Among Patients With Stroke in Control and Study Groups ADL: activity of daily living

With regard to ADL, in the control group during the pre-test, the majority of patients, 30 (100%), were partially dependent whereas during the post-test, 23% of them were partially dependent to meet their daily activities. In the study group during the pre-test, the majority of patients, 30 (100%), were partially dependent whereas during the post-test, 20% of them were partially dependent and 7% were fully independent to meet their ADL after participating in nurse-led stroke rehabilitation (Figure 6). ADL mean score during the post-test was 12.07 in the control group, and 13.23 in the study group, and the calculated p-value was 0.031, which was statistically significant at p < 0.05. Hence, the study concludes that “Nurse-Led Stroke Rehabilitation” is effective in improving the ADL scores during post-test among study group patients (Table 3).

Coping

**Figure 7:**
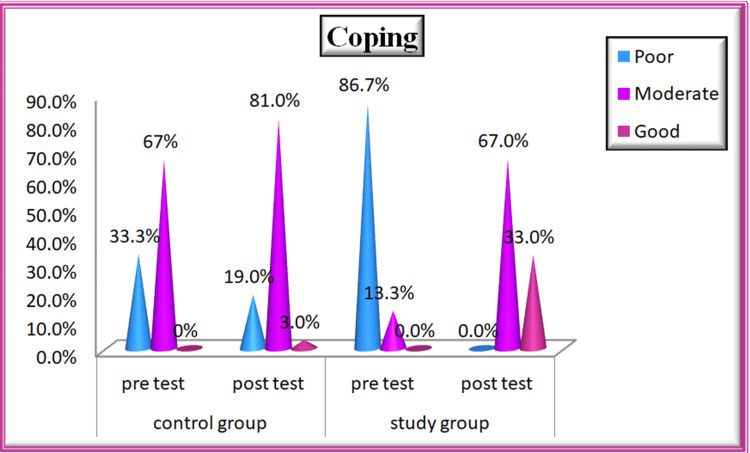
Percentage Distribution of Coping Among Patients With Stroke in Control and Study Groups

In the control group, during the pre-test, the majority of patients, 20 (66.7%), had moderate-level coping skills, while 10 patients (33.3%) had poor-level coping skills in both pre- and post-tests. In the study group, during the pre-test majority of patients, 26 (86.7%), had poor level coping skills and four patients (13.3%) had moderate level of coping skills. In the post-test, 20 patients (66.7%) showed moderate levels of coping skills, and 10 patients (33.3%) exhibited good levels of coping skills after participating in nurse-led stroke rehabilitation (Figure 7). The coping mean score during the post-test was 30.3 in the control group and 35.03 in the study group, and the calculated p-value of 0.000 which was highly significant at p < 0.01. Hence, the study concludes that “Nurse-Led Stroke Rehabilitation” is effective in improving coping skills during post-test among study group patients (Table 3).

Multiple variance analyses on stroke awareness, ADL, and coping between the control and study groups are depicted in Table 4. A comparison of stroke awareness, ADL, and coping between the groups over a period of time showed that it is significant in stroke awareness in the study group with a calculated F-value of 4.72 and p-value of 0.034, which is statistically significant at p < 0.05; in ADL, it showed that it is significant in study group with calculated F-value of 7.28 and p-value of 0.009, which is statistically significant at p < 0.05 and regarding coping the result proved that it was not statistically between the study and control groups.

**Table 4:** Multiple Variance Analysis on Stroke Awareness, ADL and Coping Between Control and Study Groups * indicates statistically significant differences; ADL: activity of daily living

Variable	Group	Mean	SD	F-value	P-value
Awareness	Control	20.5	3.2	4.72	0.034*
	Study	22.1	2.9		
ADL	Control	40.1	5.1	7.28	0.009*
	Study	43.6	4.3		
Coping	Control	15.3	2.1	3.91	0.052
	Study	16.8	2.5		

## Discussion

The present study examined 60 patients admitted with acute or hemorrhagic stroke, focusing on several clinical variables: history of diabetes mellitus, hypertension, onset of stroke, type of hemiplegia, temperature, blood sugar levels, swallowing difficulty, known cardiovascular disorders, and family history of stroke. These factors are critical for understanding stroke risk and guiding management strategies.

The first objective was to assess pre-test and post-test levels of awareness, ADL, and coping strategies among control and study group patients. In the control group, 60% had moderate awareness pretest, increasing to 70% post-test. In the study group, 50% had poor or moderate awareness pre-test, while post-test results showed 67% with moderate awareness and 33% with good awareness of stroke. These findings indicate that nurse-led stroke rehabilitation effectively improved awareness of stroke prevention and management.

In terms of ADL, all patients in the control group were classified as partially dependent at baseline. Post-intervention, 88.5% remained partially dependent, while only 11% became fully independent. In the study group, all patients were also partially dependent on the pre-test. However, following nurse-led stroke rehabilitation, 74% became partially independent and 26% fully independent. This is consistent with Sridhar et al. (2023), which highlighted the effectiveness of stroke rehabilitation programs in improving motor disability outcomes [12].

Regarding coping levels, 68% of control group patients had moderate coping at the pre-test, which improved to 81% post-test. In the study group, 86% had moderate coping after rehabilitation, with 33% achieving good coping and 67% maintaining moderate coping abilities.

The second objective was to evaluate the effectiveness of nurse-led stroke rehabilitation on stroke awareness, ADL, and coping among control and study group patients. Initially, 60% of patients were aware of stroke symptoms, while 40% had moderate awareness of stroke causes; however, awareness of prevention was low in both groups. After educational interventions, 80% of the study group improved their awareness of stroke symptoms, causes, management, and prevention, compared to a 60% improvement in the control group. The post-test mean scores were 11.7 for the control group and 13.6 for the study group, with a p-value of 0.125, indicating no statistically significant difference, though the study group had higher scores. This aligns with Devi (2024), which found that structured teaching and resources positively impacted stroke knowledge among caregivers [10].

Regarding ADL, improvements were noted in the study group, particularly in bowel and bladder control, grooming, and mobility, suggesting enhanced independence. In contrast, the control group remained dependent. The post-test mean scores for ADL were 12.07 in the control group and 13.23 in the study group, with a statistically significant p-value of 0.031. These findings support Aljohani et al. (2024), which highlighted the effectiveness of nurse-led programs in improving motor disability outcomes [5].

For coping strategies, the study group showed significant improvements in approach coping, avoidance coping, altering consciousness, and seeking support. The control group exhibited minimal change. The post-test mean scores were 30.3 for the control group and 35.03 for the study group, with a highly significant p-value of 0.000. This is consistent with García et al. (2018), emphasizing the need for evidence-based interventions to address psychosocial needs post-stroke [19].

Limitations of the study include its quasi-experimental design and the use of purposive sampling, along with a short data collection period of four weeks. The stroke patients were matched with an mRS score of less than 3, which indicates moderate disability, age between 35 and 60 years (in India, a significant portion of stroke occurs among individuals under 40 years of age), and the goodness-of-fit test was determined through the chi-square test.

## Conclusions

People typically gather information about stroke from family and friends, leading to a generally poor awareness of stroke prevention. A multidisciplinary supervised program has proven more effective than self-monitoring in stroke rehabilitation. Key components of stroke rehabilitation include educating patients and families, addressing stroke-related complications, and preventing recurrent strokes. Positive predictors of stroke outcomes include having a supportive spouse and the ability to perform daily activities independently. Following an ischemic stroke, more intensive strategies are required to encourage physical activity, as patients often lose their coping abilities. Therefore, rehabilitation nurses should emphasize coping strategies to help improve patients' functional abilities and quality of life. Research supports the idea that stroke patients involved in nurse-led rehabilitation programs show significant improvements in awareness, daily functioning, and coping strategies. The findings of this study underscore the effectiveness of such rehabilitation efforts in enhancing these critical areas, illustrating the substantial benefits of nurse-led stroke rehabilitation for better recovery outcomes.

## Figures and Tables

**Figure 8:**
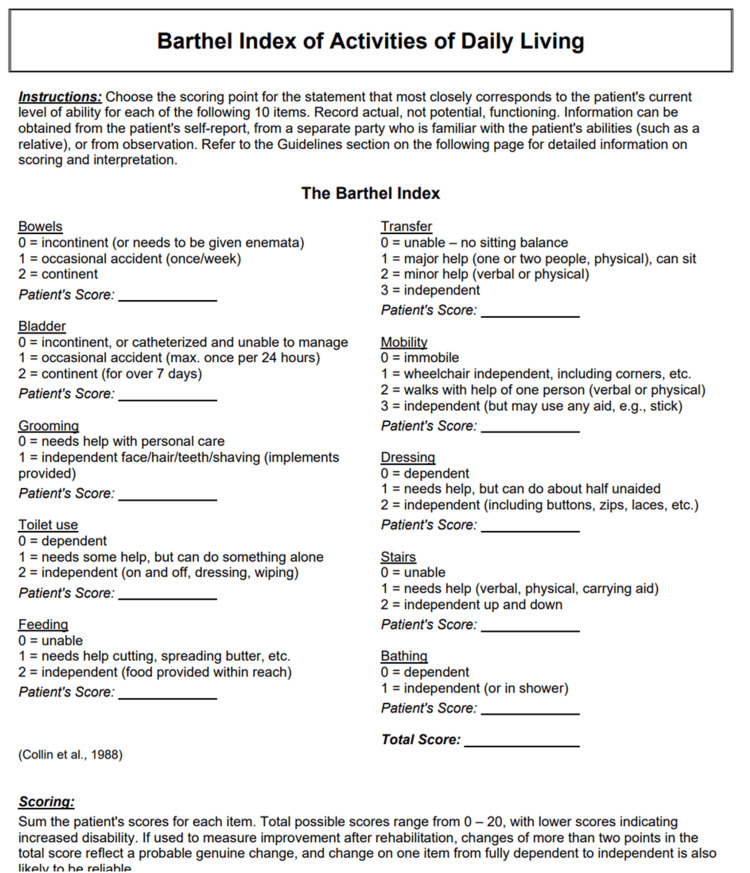
Barthel Index of Activities of Daily Living

## Appendices

Section A - Demographic Variables

1. Age

a) 30-40 years

b) 41-50 years

c) Above 50 years

2. Gender

a) Male

b) Female

3. Education

a) Profession

b) Diploma

c) Higher secondary school

d) High school

e) Illiterate 

4. Marital status

a) Married

b) Unmarried

c) Widower

5. Occupation

b) employ

d) Unemployed

6. Monthly income

a) 5120-7680

b) 7681-10,240

c) 10,241-20,481

d) ≥20,482

e) None

7. Type of family

a) Joint family

b) Nuclear family

c) Extended family

Section B: Clinical Variables

1. History of diabetes mellitus

a) Yes

b) No

2. History of hypertension

a) Yes

b) No

3. Onset of stroke symptoms admitted to ER

a) Within 4 hrs

b) Within 6 hrs

c) Within 12 hrs

4. Type of Hemiplegia ___________________

5. GCS ______________

6. Muscle power _______________

7. Temperature _____________

8. Blood Suger ______________

9. Swallowing difficulty ________________

10. Known history of cardiovascular disorders

a) Yes

b) No

11. Family history of stroke

a) Yes

b) No

Section C: Stroke Knowledge Assessment Tool

**Table 5:** Stroke Knowledge Assessment Tool 1: strongly agree, 2: agree, 3: undecided, 4: disagree, 5: strongly disagree

		Answer
Knowledge		
K1	Smoking is a risk factor for stroke	
K2	Stroke is commoner than breast cancer	
K3	Chest pain is a common symptom of stroke	
K4	Loss of vision and vertigo can be secondary to stroke	
K5	Previous transient ischaemic attacks (TIAs) increase future stroke risk	
K6	Stroke affects the heart	
K7	Difficulty to talk or understand speech should raise suspicion of stroke	
K8	Stroke is a rare cause of long-term disability	
K9	Stroke is more common in females	
K10	Diabetes increases the risk of stroke	
K11	Headache may occasionally be secondary to stroke	
K12	Sudden loss of sensation is not related to stroke	
K13	Stroke is always characterised by symptoms	
K14	Muscle or limb power is not affected in stroke	
K15	The acronym ‘FAST’ in the ‘Act FAST’ campaign stands for Face, Arms, Speech and Time	
K16	Patients who have suffered a stroke should be advised to adopt a sedentary lifestyle	
K17	Mass media adverts and educational leaflets on stroke are effective in increasing stroke awareness and education	

 Section D: Barthel Index of Activities of Daily Living

Section E: Brief Cope

Brief COPE (Carver, 1997).

You have done really well - thank you. These next items deal with ways you've been coping with the stress in your life. The stress issue is the ‘it’ in some of the items! There are many ways to try to deal with problems. These items ask what you've been doing to cope with present stresses. Each item says something about a particular way of coping and please avoid answering on the basis of whether how you’ve been coping seems to be working or not-just whether or not you're doing it. Use these response choices and try to rate each item separately in your mind from the others. Make your answers as true FOR YOU as you can.

Coding categories:

1 = I haven't been doing this at all

2 = I've been doing this a little bit

3 = I've been doing this a medium amount

4 = I've been doing this a lot

1.   I've been turning to work or other activities to take my mind off things.

2.   I've been concentrating my efforts on doing something about the situation I'm in.

3.   I've been saying to myself "this isn't real."

4.   I've been using alcohol or other drugs to make myself feel better.

5.   I've been getting emotional support from others.

6.   I've been giving up trying to deal with it.

7.   I've been taking action to try to make the situation better.

8.   I've been refusing to believe that it has happened.

9.   I've been saying things to let my unpleasant feelings escape. *

10.   I’ve been getting help and advice from other people.

11.   I've been using alcohol or other drugs to help me get through it.

12.   I've been trying to see it in a different light, to make it seem more positive.

13.   I’ve been criticizing myself.

14.   I've been trying to come up with a strategy about what to do.

15.   I've been getting comfort and understanding from someone.

16.   I've been giving up the attempt to cope.

17.   I've been looking for something good in what is happening.

18.   I've been making jokes about it.

19.   I've been doing something to think about it less, such as going to movies, watching TV, reading, daydreaming, sleeping, or shopping.

20.   I've been accepting the reality of the fact that it has happened.

21.   I've been expressing my negative feelings.

22.   I've been trying to find comfort in my religion or spiritual beliefs.

23.   I’ve been trying to get advice or help from other people about what to do.

24.   I've been learning to live with it.

25.   I've been thinking hard about what steps to take.

26.   I’ve been blaming myself for things that happened.

27.   I've been praying or meditating.

28.   I've been making fun of the situation.

You can use the 14 types of coping the Carver identifies but Carver recommends users to carry out their own factor analysis to decide the best grouping of items. I did this with approximately 300 nursing students and identified that the 28 items can be grouped into four types of coping. If you are opting for running regressions then it makes sense to use fewer factors to make the regression more manageable. The four types of coping I identified were:

Approach coping: 1, 2, 7, 12, 14, 17, 20, 24, 25

Avoidance coping: 3, 6, 8, 13, 16, 18, 19, 26, 28

Altering consciousness: 4, 11, 22, 27

Seeking support: 5, 10, 15, 21, 23

* Item 9 did not load onto any of the four types of coping and so was excluded.

## References

[1] GBD 2019 Stroke Collaborators (2021). Global, regional, and national burden of stroke and its risk factors, 1990-2019: a systematic analysis for the Global Burden of Disease Study 2019. Lancet Neurol.

[2] Grefkes C, Fink GR (2020). Recovery from stroke: current concepts and future perspectives. Neurol Res Pract.

[3] Virani SS, Alonso A, Benjamin EJ (2020). Heart disease and Stroke Statistics-2020 update: a report from the American Heart Association. Circulation.

[4] Pu L, Wang L, Zhang R, Zhao T, Jiang Y, Han L (2023). Projected global trends in ischemic stroke incidence, deaths and disability-adjusted life years from 2020 to 2030. Stroke.

[5] Aljohani KA, Fadlalmola HA, Fadila DES (2024). Effects of the nurse-led program on disability improvement in patients with stroke: a systematic review and meta-analysis. J Disabil Res.

[6] Wang J, Zhang Y, Chen Y (2021). Effectiveness of rehabilitation nursing versus usual therapist-led treatment in patients with acute ischemic stroke: a randomized non-inferiority trial. Clin Interv Aging.

[7] Verberne DPJ, Kroese MEAL, Staals J, Ponds RWHM, van Heugten CM (2022). Nurse-led stroke aftercare addressing long-term psychosocial outcome: a comparison to care-as-usual. Disabil Rehabil.

[8] Liang J, Luo C, Ke S, Tung TH (2023). Stroke related knowledge, prevention practices and associated factors among stroke patients in Taizhou, China. Prev Med Rep.

[9] Jones SP, Jenkinson AJ, Leathley MJ, Watkins CL (2010). Stroke knowledge and awareness: an integrative review of the evidence. Age Ageing.

[10] Devi B (2024). Efficacy of stroke empowerment program on stroke knowledge among caregivers with stroke survivors. Int J Nutr Pharmacol Neurol Dis.

[11] Kumari R, Gupta RK, Shalli Shalli, Bahl R, Langer B (2024). Assessment of dependency in activities of daily living (ADL) and its predictors: a cross-sectional study among the elderly rural population in a sub-Himalayan UT of India. Indian J Community Med.

[12] Sridhar T, Varghese LE, Hasharudeen A (2024). A study evaluating the aspects of stroke-specific quality of life and severity in hypertensive stroke patients. Asian J Pharm Res Health Care.

[13] Angel M, Rani SR (2023). Stress and coping strategies among the families of patients admitted with stroke in selected hospitals. TRR.

[14] Clare CS (2020). Role of the nurse in acute stroke care. Nurs Stand.

[15] Clare CS (2018). Role of the nurse in stroke rehabilitation. Nurs Stand.

[16] Kamalakannan S, Gudlavalleti AS, Gudlavalleti VS, Goenka S, Kuper H (2017). Incidence & prevalence of stroke in India: a systematic review. Indian J Med Res.

[17] Grech R, Grech P (2021). The Stroke Knowledge Assessment Tool (SKAT): development, reliability and validity. J Med Health Sci.

[18] Collin C, Wade DT, Davies S, Horne V (1988). The Barthel ADL Index: a reliability study. Int Disabil Stud.

[19] García FE, Barraza-Peña CG, Wlodarczyk A, Alvear-Carrasco M, Reyes-Reyes A (2018). Psychometric properties of the Brief-COPE for the evaluation of coping strategies in the Chilean population. Psicol Reflex Crit.

